# Evaluation of health behaviors and Type D personality traits among physiotherapy students in relation to musculoskeletal disorder symptoms

**DOI:** 10.3389/fpsyg.2025.1710640

**Published:** 2026-01-14

**Authors:** Magdalena Gębska, Łukasz Pałka, Dominik Chmielewski, Katarzyna Weber-Nowakowska, Piotr Seweryn, Bartosz Dalewski

**Affiliations:** 1 Department of Rehabilitation Musculoskeletal System, Pomorski Uniwersytet Medyczny w Szczecinie, Szczecin, Poland; 2 Private Practitioner, Żary, Poland; 3 Department of Experimental Dentistry, Uniwersytet Medyczny im Piastow Slaskich we Wroclawiu, Wrocław, Poland; 4 Department of Dental Prosthetics, Pomeranian Medical University, Szczecin, Poland

**Keywords:** depression, healthy behaviors, musculoskeletal system, physiotherapy students, stress, Type D personality

## Abstract

**Introduction:**

Existing literature lacks studies evaluating the role of health behaviors and Type D personality in relation to musculoskeletal disorder symptoms. This study is the first to analyze these factors, specifically targeting a group at heightened risk of motor system disorders in their future professional roles—physiotherapy students.

**Aim:**

To assess health behaviors, prevalence of Type D personality, perceived stress levels, and depression in the context of musculoskeletal disorder symptoms among physiotherapy students.

**Materials and methods:**

The study included 100 students (2nd- and 4th-year) and utilized a custom survey alongside standardized diagnostic tools: the Health Behavior Inventory, DS14, PSS10, and the Beck Depression Inventory.

**Results:**

Men reported higher mean scores of unhealthy behaviors than women (Cohen’s d 0.72, moderate effect size; *p* = 0.006). A stronger Type D personality was associated with a decrease in healthy behaviors, while increased social inhibition correlated with a reduction in unhealthy behaviors. No statistically significant relationships were found between unhealthy behaviors and the presence of musculoskeletal disorder symptoms. Statistically significant positive relationships were found only between negative emotionality and the number of pain complaints (*r* = 0.20; *p* = 0.042; 95% CI [0.01, 0.39]) and the general level of musculoskeletal pain (*r* = 0.20; *p* = 0.048; 95% CI [<0.01, 0.38]). Perceived stress and depressive symptoms contributed to a reduction in healthy behaviors (0.30 < *r* < 0.50). Additionally, higher levels of Type D personality, particularly in negative emotionality and social inhibition dimensions, were associated with increased stress and greater severity of depressive symptoms (*r* > 0.50).

**Conclusion:**

Our findings show that gender is associated with health behaviors among students, with male physiotherapy students exhibiting more unhealthy behaviors than female students. Type D personality is associated with health behaviors and may be important for the frequency and severity of musculoskeletal pain symptoms.

## Introduction

Health behaviors are reactive, habitual, or intentional actions that are fundamentally connected to health, grounded in both objective knowledge and subjective beliefs about well-being (Conner and Norman, 2017). These behaviors include both healthy and unhealthy actions. Healthy behaviors encompass a balanced diet, proper weight regulation, adequate physical activity and rest (relaxation, sleep), stress management, maintenance of social bonds, mental and physical hygiene, preventive behaviors, regular medical check-ups, and adherence to medical recommendations (Lippke et al., 2012; Jiang et al., 2024). Unhealthy behaviors involve risky driving, unsafe sexual practices, smoking, excessive alcohol consumption, and the use of toxic or psychoactive substances (Lippke et al., 2012; Jiang et al., 2024). Some of them, especially excessive alcohol use and psychoactive substance consumption, are prevalent in the population of university students. As a healthy lifestyle is widely recognized as a preventive factor against numerous lifestyle-related diseases, including those affecting the musculoskeletal system, healthy behaviors should be instilled from an early age, both within the family and educational environments (Jiang et al., 2024).

Health promotion initiatives are supported by a range of institutions and organizations. It is crucial to highlight that this collective societal effort to promote and safeguard health should be undertaken by professionals trained specifically for this purpose (Tannahill, 2008; Cianciara et al., 2010). Among these professionals are various roles, including physicians, nurses, and psychologists. Physiotherapy graduates, in particular, are also expected to serve as health promoters; due to their specialized knowledge, they are anticipated to model appropriate health behaviors (Kubińska et al., 2020).

However, it is important to recognize that physiotherapy students, similar to other demographic groups, are also subject to numerous stressors, including the demands of adjusting to a new lifestyle, managing unfamiliar responsibilities, meeting academic requirements, and coping with a heavy workload—all of which can hinder their ability to maintain a healthy lifestyle (Kubińska et al., 2020). Upon entering the workforce, these graduates will encounter various occupational hazards, both physical and mental. One of the primary occupational risks for physiotherapists involves mechanical strain on the musculoskeletal system, due to frequent lifting of patients, maintaining constrained body positions, and repetitive, asymmetrical load from standard therapeutic techniques (Chen et al., 2022). Thus, it is essential to evaluate the occurrence of musculoskeletal symptoms in future physiotherapists, as implementing preventive strategies, ergonomic training, and early intervention could mitigate potential long-term musculoskeletal health issues.

This perspective led us to examine the associations between health behaviors and musculoskeletal symptoms among physiotherapy students. As advocates of healthy living, they are expected to set a positive example for their patients by modeling healthy behaviors. Additionally, the study sought to determine the role of personality type in influencing students’ health behaviors. In this context, the concept of stress-prone, or Type D, personality may be significant. Defined by Johan Denollet in 1995, Type D personality comprises two dimensions: negative emotionality and social inhibition (Niestrój-Jaworska et al., 2022). The former reflects a tendency to consistently experience negative emotions, while the latter indicates a predisposition to suppress these emotions in social interactions. Type D personality has been identified as a substantial predictor of coronary artery disease and related mortality (Howard and Hughes, 2012). Furthermore, Polish studies suggest a correlation between Type D personality and various health conditions, including cardiovascular disease and other somatic disorders, such as cancer, peptic ulcers, skin conditions, and temporomandibular joint disorders (Raykh et al., 2020; Kim et al., 2021; Akram et al., 2018; Jordi et al., 2021; Gębska et al., 2022).

The available literature lacks studies that connect Type D personality with health behaviors and musculoskeletal disorder symptoms in physiotherapy students. However, previous research has shown that individuals with Type D personality are less likely to exercise, maintain a healthy diet, adhere to prescribed medications, and attend regular medical check-ups compared to those with other personality types (Wiencierz and Williams, 2016; Wang et al., 2020; Booth and Williams, 2015). Furthermore, this personality type is prone to higher levels of emotional stress (Stevenson and Williams, 2014).

This study aimed to evaluate health behaviors, the prevalence of Type D personality, perceived stress, and depression in relation to musculoskeletal disorder symptoms among physiotherapy students, who, in their future roles, will serve as role models in terms of healthy lifestyle for their patients.

The following research questions were formulated:

1. Are gender and year of study associated with students’ health behavior?
2. Is there a relationship between health and unhealthy behaviors and the severity of Type D personality?
3. Is there a relationship between healthy and unhealthy behaviors and the presence of musculoskeletal disorder symptoms?
4. Is there a relationship between the severity of Type D personality and the presence of musculoskeletal disorder symptoms?
5. Is there a relationship between healthy and unhealthy behaviors and the levels of perceived stress and depression severity?
6. Is there a relationship between the severity of Type D personality and the levels of perceived stress and depression severity?

## Materials and methods

The research project was approved by the Bioethics Committee of the Pomeranian Medical University in Szczecin on September 9, 2022 (KB.006.157.2022/Z-11207). All participants provided written informed consent to participate in the study.

The research was conducted from October 2024 to June 2025.

The study group included male and female physiotherapy students in their 2nd and 4th years (*n* = 100). The research was conducted at the Department of Motor System Rehabilitation at the Pomeranian Medical University, following the completion of class sessions, with the instructors’ consent and after scheduling an agreed-upon time. Students were informed of the study’s purpose, and participation was voluntary. The study session lasted 30 min, during which students were asked to complete an anonymous self-assessment questionnaire.

### Research tools

1. Our own questionnaire consisting of personal details (age, gender, weight, height, field of study and year of study) and single- and multiple-choice questions regarding the occurrence of symptoms of musculoskeletal disorders, physical activity, unhealthy activities, and the occurrence of comorbidities.
2. Health Behavior Inventory (HBI). The HBI questionnaire by Juczyński (2009) was used to examine behaviors related to a healthy lifestyle. It consists of 25 statements describing various health-related behaviors, to which the respondent responds by marking the frequency of a given behavior on a 5-point scale (1—almost never, 2—rarely, 3—from time to time, 4—often, 5—almost always). The results are categorized into 4 scales measuring individual aspects of health behaviors: Positive Mental Attitudes (PMA), Preventive Behaviors (PBE), Proper Eating Habits (HH) and Health Practices (HP). In addition to the average results in a given scale, the overall IZZ result is also taken into account as the sum of all the results from the individual scales (Niestrój-Jaworska et al., 2022). The higher the score, the greater the level of health care and the more frequent health-promoting behaviors (Niestrój-Jaworska et al., 2022). The author of the questionnaire provides sufficient reliability for the overall IZZ result (*α* = 0.85), and for the individual scales (Cronbach’s alpha coefficient ranged from 0.60 to 0.65; Juczyński, 2009). In the present study, the internal consistency was (Cronbach’s alpha) 0.88 for the overall HBI result, and 0.74, 0.67; 0.73 and 0.59 for the scales: HH, PBE, PMA and HP, respectively.
3. Psychological Questionnaire DS14 (Type-D scale). For assessing the presence of a stressful personality in this study the authors used the validated Polish adaptation of DS-14 (Ogińska-Bulik and Juczyński, 2009). It consists of 7 related to the tendency to experience negative emotions and 7 tendencies to refrain from expressing these emotions. Each statement is rated on a scale from 0 (false) to 4 (true). The theoretical range of scores for each dimension is 0 to 28 points. Classification to type D requires obtaining at least 10 points in each of the two dimensions, i.e., NE (negative emotionality) and SI (social inhibition). A high score on the NE dimension (≥10) indicates strong emotional reactivity, increased tension and susceptibility to stress, and a tendency to worry. A low score (<10) indicates emotional stability and a relatively low level of negative emotions. A high score on the SI dimension (≥10) indicates a tendency to withdraw, a reluctance to express emotions, and tension in relationships. A low score on the SI dimension (<10) indicates ease in relationships and ease in self-expression (Ogińska-Bulik and Juczyński, 2009). Cronbach’s alpha for the DS14 was 0.86.
4. Perceived Stress Scale (PSS-10). For assessing the stress level in this study, we used validated Polish adaptation of PSS10 (Juczyński and Ogińska-Bulik, 2009). It contains 10 questions about different subjective feelings related to personal problems and events, behaviors and ways of coping. The respondents provided their answers by entering the correct number (0—never, 1—almost never, 2—sometimes, 3—quite often, 4—very often). The overall score on the scale is the total of all points, the theoretical distribution of which is from 0 to 40. The higher the score, the greater the severity of the perceived stress. The general indicator after conversion to standardized units is interpreted according to the properties characterizing the sten scale (is a scale of psychological test normalized so that the population mean is 5.5 and the standard deviation is 2. The scale has 10 units). Scores in the range 1–4 sten (0–13 points) are treated as low, and in the range 7–10 sten (20–40 points) as high. Results between 5 and 6 sten (14–19 points) were considered average. A person with a high score may experience: a sense of lack of control, tension and difficulty relaxing, an accumulation of daily challenges, and easier emotional overload. A person with a low score has developed effective coping strategies and a sense of control over the situation (Juczyński and Ogińska-Bulik, 2009). Cronbach’s alpha for the PSS-10 was 0.74.
5. Beck Depression Inventory (BDI). Advantages of the inventory are its high internal consistency, high content validity, validity in differentiating between depressed and nondepressed subjects, sensitivity to change. The original version has proven and satisfactory psychometric properties, while the data on the Polish translation are still only preliminary, despite very frequent use in practice and research (Parnowski and Jernajczyk, 1977). BDI consists of 21 questions. The participants can choose one of four answers to each question. Each answer is assigned a value of 0 to 3 points. The theoretical range of scores for each dimension is 0 to 63 points. A score of 0 to 13 indicates minimal or no symptoms of depression, meaning a low level of sadness, dejection, or even a lack thereof. This range is typically interpreted as minimal depressive symptom severity. A score of 14 to 19 indicates mild symptoms of depression (may include a decreased sense of well-being, low mood, and decreased energy), and is typically interpreted as mild depressive symptom severity. A score of 20 to 28 indicates moderate symptoms of depression (severe depression, decreased motivation, difficulty concentrating, changes in appetite or sleep). This range is typically interpreted as moderate depressive symptom severity. A score of 29 to 63 indicates severe symptoms of depression (a marked, prolonged low mood, negative thoughts about oneself, and difficulty with daily functioning), and is typically interpreted as severe depressive symptom severity (Parnowski and Jernajczyk, 1977). Cronbach’s alpha for the BDI was 0.84.

### Statistical analysis

Statistical analysis was conducted based on the collected data. Statistical analyses were performed using IBM SPSS Statistics 30. Descriptive statistics were calculated, and the Shapiro–Wilk test was applied to assess variable distributions and verify the normality assumption. Pearson’s correlation analysis was conducted to examine relationships between two quantitative variables, and the Mann–Whitney test was used to compare two unequal groups in terms of quantitative variable levels. A significance level of *α* = 0.05 was adopted for this analysis. The Shapiro–Wilk test indicated statistically significant deviations from normality for all musculoskeletal disorder symptoms, unhealthy behaviors, and depression levels. Despite this, the skewness of these variables did not exceed an absolute value of 2, indicating only slight asymmetry (Field, 2018). Therefore, parametric tests were considered appropriate for further analysis, provided all other assumptions were met.

## Results

### Group characteristics

Table 1 presents the demographic and clinical characteristics of the group.

**Table 1:** Group characteristics.

Variable	Variable	*n*(%)	*M*(*SD*)
Gender	Female	66 (66.0%)	-
	Male	34 (34.0%)	-
Field of study	Physiotherapy	100 (100.0%)	-
Year of study	2	69 (69.0%)	-
	4	31 (31.0%)	-
Physical activity	No	20 (20.0%)	-
	Yes	80 (80.0%)	-
Frequency of physical activity	I do not practice	20 (20.0%)	-
	Less than 3 times a week	40 (40.0%)	-
	3–4 times a week	35 (35.0%)	-
	Daily	5 (5.0%)	-
Health assessment	Poor	1 (1.0%)	-
	Fair	29 (29.0%)	-
	Good	52 (52.0%)	-
	Very good	18 (18.0%)	-
Chronic diseases	No	67 (67.0%)	-
	Yes	33 (33.0%)	-
Age		-	20.90 (1.12)
Body weight		-	69.74 (15.46)
Height		-	172.37 (9.80)
BMI		-	23.26 (3.45)

n, number of observations; %, percent; M, mean; SD, standard deviation.

In the initial analysis, we examined the distributions of quantitative variables by calculating basic descriptive statistics and using the Shapiro–Wilk test to assess normality. The results are presented in Table 2.

**Table 2:** Basic descriptive statistics of the studied variables with the Shapiro–Wilk test (*N* = 100).

Variable	*M*	*Me*	*SD*	*Sk.*	*Kurt.*	*Min.*	*Max.*	*W*	*p*
Symptoms of musculoskeletal disorders									
Intensity of pain complains	0.99	0.89	0.63	0.77	1.05	0.00	3.44	0.96	0.002
Number of pain complains	5.79	5.00	4.89	1.02	0.49	0.00	20.00	0.90	<0.001
Level of pain complains	7.63	3.28	9.54	1.92	4.16	0.00	48.22	0.77	<0.001
Anti-health behaviors									
Anti-health behaviors	2.46	2.00	2.24	1.46	2.54	0.00	11.00	0.85	<0.001
DS-14									
Intensity of type D personality	26.23	25.50	9.95	−0.09	−0.18	2.00	47.00	0.99	0.317
Negative emotionality (NE)	15.00	15.00	6.34	−0.24	−0.37	1.00	28.00	0.98	0.173
Social inhibition (SI)	11.23	11.00	5.31	0.24	−0.54	1.00	25.00	0.98	0.167
PSS-10									
Level of perceived stress	21.00	22.00	6.63	−0.36	0.02	4.00	34.00	0.98	0.083
BDI									
Level of depression	12.75	10.00	9.41	1.00	0.69	0.00	42.00	0.92	<0.001
HBI									
General intensity of health behaviors	73.57	74.00	14.12	0.42	0.46	44.00	115.00	0.98	0.143
Proper eating habits	18.66	19.00	5.03	0.06	−0.17	7.00	30.00	0.99	0.383
Preventive behaviors	18.40	18.00	4.68	0.12	−0.23	8.00	30.00	0.99	0.344
Positive mental attitude	18.62	19.00	4.52	0.05	0.22	8.00	30.00	0.99	0.472
Health practices	17.89	18.00	3.94	0.23	0.21	9.00	29.00	0.99	0.331

M, mean; Me, median; SD, standard deviation; Sk., skewness; Kurt., kurtosis; Min., minimum value; Max., maximum value; W, result of the Shapiro–Wilk test; *p*, statistical significance for the Shapiro–Wilk test.

We then examined whether gender and year of study were associated with healthy and unhealthy behaviors in the study group. Comparisons were made between males and females, as well as between 2nd- and 4th-year students, with respect to the intensity of healthy behaviors and the frequency of unhealthy behaviors. Due to the statistically significant imbalance in group sizes [sex: χ^2^(1) = 10.24, *p* < 0.001; year of study: χ^2^(1) = 14.44, *p* < 0.001], the non-parametric Mann–Whitney test was used. The results are displayed in Table 3.

**Table 3:** Comparison of women and men and 2nd year students with 4th year students in terms of the intensity of healthy and anti-health behaviors (*N* = 100).

Dependent variable	Group	*M*	*SD*	*t*	*df*	*p*	*Cohen’s d*
Anti-health behaviors	Women (*n* = 66)	1.94	1.68	2.91^a^	45.42	0.006	0.72
	Men (*n* = 34)	3.47	2.82				
Overall intensity of health behaviors	Women (*n* = 66)	74.52	13.89	0.93	98	0.354	0.20
	Men (*n* = 34)	71.74	14.59				
Proper eating habits	Women (*n* = 66)	19.32	5.02	1.85	98	0.068	0.39
	Men (*n* = 34)	17.38	4.86				
Preventive behaviors	Women (*n* = 66)	18.77	4.52	1.11	98	0.269	0.23
	Men (*n* = 34)	17.68	4.96				
Positive mental attitude	Women (*n* = 66)	18.47	4.26	−0.46	98	0.646	0.10
	Men (*n* = 34)	18.91	5.05				
Health practices	Women (*n* = 66)	17.95	3.76	0.23	98	0.821	0.05
	Men (*n* = 34)	17.76	4.32				
Anti-health behaviors	2nd year (*n* = 69)	2.61	2.38	1.08^a^	71.89	0.285	0.21
	4^th^ year (*n* = 31)	2.13	1.89				
Overall intensity of health behaviors	2nd year (*n* = 69)	72.09	14.02	−1.58	98	0.118	0.34
	4^th^ year (*n* = 31)	76.87	14.00				
Proper eating habits	2nd year (*n* = 69)	18.45	5.43	−0.70^a^	76.41	0.488	0.13
	4^th^ year (*n* = 31)	19.13	4.03				
Preventive behaviors	2nd year (*n* = 69)	17.97	4.52	−1.37	98	0.173	0.30
	4^th^ year (*n* = 31)	19.35	4.96				
Positive mental attitude	2nd year (*n* = 69)	18.04	4.50	−1.93	98	0.057	0.42
	4^th^ year (*n* = 31)	19.90	4.38				
Health practices	2nd year (*n* = 69)	17.62	3.75	−1.01	98	0.315	0.22
	4^th^ year (*n* = 31)	18.48	4.33				

a The result of Levene’s test was statistically significant - the result was reported with Welch’s correction.

n, number of observations; M, mean; SD, standard deviation; t, test statistic; df, degrees of freedom; *p*, statistical significance; Cohen’s d-effect size.

The analysis revealed a statistically significant difference in unhealthy behaviors only between men and women, with men reporting higher levels of unhealthy behaviors than women. The observed effect was moderate (0.50 < *Cohen’s d* < 0.80). There were no statistically significant gender differences for other variables, nor were there significant differences between second- and fourth-year students in the intensity of unhealthy and healthy behaviors.

The next stage of analysis explored associations between healthy behaviors, unhealthy behaviors, Type D personality traits, musculoskeletal disorder symptoms, and levels of perceived stress and depression. A series of Pearson’s correlation analyses were performed, and the results are presented in Table 4.

**Table 4:** Correlation of health behaviors, unhealthy behaviors, type D personality severity, symptoms of musculoskeletal disorders, level of perceived stress and depression (*N* = 100).

Variable	2.	3.	4.	5.	6.	7.	8.	9.	10.	11.	12.	13.	14.
1. Anti-health behaviors	−0.34***	−0.26**	−0.27**	−0.24*	−0.30**	−0.13	−0.04	−0.21*	−0.01	−0.15	−0.06	0.10	0.16
2. General intensity of health behaviors	-	0.76***	0.80***	0.80***	0.75***	−0.31**	−0.29**	−0.23*	0.05	0.04	0.01	−0.33***	−0.37***
3. Proper eating habits		-	0.49***	0.38***	0.43***	−0.13	−0.09	−0.13	0.05	0.10	0.07	−0.14	−0.16
4. Preventive behaviors			-	0.57***	0.39***	−0.16	−0.12	−0.15	0.06	0.02	0.02	−0.16	−0.16
5. Positive mental attitude				-	0.57***	−0.46***	−0.48***	−0.28**	0.02	−0.09	−0.08	−0.43***	−0.49***
6. Health practices					-	−0.23*	−0.25*	−0.13	0.02	0.11	0.02	−0.34***	−0.35***
7. Intensity of type D personality						-	0.88***	0.82***	0.03	0.16	0.13	0.68***	0.59***
8. Negative emotionality (NE)							-	0.45***	0.11	0.20*	0.20*	0.76***	0.63***
9. Social inhibition (SI)								-	−0.08	0.05	0.01	0.37***	0.34***
10. Intensity of pain complaints									-	0.62***	0.82***	0.19	0.26**
11. Number of pain complaints										-	0.89***	0.21*	0.33***
12. Level of pain complaints											-	0.24*	0.33***
13. Level of experienced stress												-	0.69***
14. Level of depression													-

*p < 0.050; **p < 0.010; ***p < 0.001.

First, the relationships between health and anti-health behaviors and the severity of type D personality were analyzed. The obtained results indicated the occurrence of statistically significant negative relationships between anti-health behaviors and social inhibition (*r* = −0.21; *p* = 0.040; 95% CI [−0.39, −0.01]), the general intensity of health behaviors and social inhibition (*r* = −0.23; *p* = 0.024; 95% CI [−0.40,-0.03]), and positive psychological attitude and social inhibition (*r* = −0.28; *p* = 0.004; 95% CI [−0.46, −0.09]), as well as between the general intensity of health behaviors and the general severity of type D personality (*r* = −0.31; *p* = 0.002; 95% CI [−0.48, −0.12]) and negative emotionality (*r* = −0.29; *p* = 0.003; 95% CI[−0.46, −0.11]), positive mental attitude and the general severity of type D personality (*r* = −0.46; *p* < 0.001; 95% CI [−0.60, −0.29]) and negative emotionality (*r* = −0.48; *p* < 0.001; 95% CI [−0.62, −0.32]), and health practices and the general severity of type D personality (*r* = −0.23; *p* = 0.021; 95% CI [−0.41, −0.04]) and negative emotionality (*r* = −0.25; *p* = 0.012; 95% CI [−0.43, −0.06]). This suggests that as Type D personality traits and negative emotionality increased, levels of overall healthy behaviors, positive affect, and health practices decreased. Additionally, as levels of social inhibition increased, unhealthy behaviors, overall healthy behaviors, and positive affect declined. However, most correlations were weak (*r* < 0.30), with only moderate correlations observed between the overall intensity of Type D personality traits and both overall healthy behaviors and positive affect, as well as between negative emotionality and positive affect (0.30 < *r* < 0.50).

Next, the relationships between the severity of Type D personality and the occurrence of musculoskeletal symptoms were verified. The analysis revealed statistically significant positive relationships only between negative emotionality and the number of pain symptoms (*r* = 0.20; *p* = 0.042; 95% CI [0.01, 0.39]) and the overall level of musculoskeletal pain (*r* = 0.20; *p* = 0.048; 95% CI [<0.01, 0.38]). This indicates that variations in the frequency of unhealthy behaviors or the intensity of healthy behaviors, both overall and across specific dimensions, were not associated with changes in the frequency, intensity, or overall level of musculoskeletal pain symptoms.

The next step was to analyze the relationships between healthy and unhealthy behaviors and the level of perceived stress and depression. The obtained results indicated the occurrence of statistically significant negative relationships between the level of perceived stress and the general intensity of health-related behaviors (*r* = −0.33; *p* < 0.001; 95% CI [−0.50, −0.15]), positive mental attitude (*r* − 0.43; *p* < 0.001; 95% [−0.58; −0.25]) and health practices (*r* = −0.34; *p* < 0.001; 95% CI [−0.50, −0.16]) as well as between the level of depression and the general intensity of health-related behaviors (*r* = −0.37; *p* < 0.001; 95% [−0.53, −0.18]), positive mental attitude (*r* − 0.49; *p* < 0.001; 95% CI [−0.63, −0.33]) and health practices. Health (*r* = −0.36; *p* < 0.001; 95% CI [−0.52, −0.17]). This indicates that as perceived stress and depressive symptoms increased, the intensity of overall healthy behaviors, positive affect, and health practices decreased. Notably, these correlations were moderate (0.30 < *r* < 0.50).

The last part of this stage of analysis was to verify whether there were any relationships between the severity of type D personality and the level of perceived stress and the severity of depression. The analysis revealed statistically significant positive relationships between the level of perceived stress and the severity of type D personality in general (*r* = 0.68; *p* < 0.001; −95% CI [0.56, 0.78]) and in the dimensions of negative emotionality (*r* = 0.76; *p* < 0.001; −95% [0.67, 0.83]) and social inhibition (*r* = 0.37; *p* < 0.001; −95% CI [0.19, 0.53]), as well as between the level of depression and the severity of type D personality in general (*r* = 0.59; *p* < 0.001; −95% CI [0.44, 0.70]) and in the dimensions of negative emotionality (*r* = 0.63; *p* < 0.001; −95% CI [0.50, 0.74]) and social inhibition (*r* = 0.34; *p* < 0.001; −95% CI [0.16, 0.51]). This suggests that as overall Type D personality intensity, negative emotionality, and social inhibition increased, levels of perceived stress and depressive symptoms also rose. Most observed correlations were strong (*r* > 0.50), with only moderate correlations between social inhibition and both stress and depression (0.30 < *r* < 0.50).

## Discussion

The aim of this study was to highlight the need for both quantitative and qualitative research on health behaviors among medical students, as the current body of literature on this topic remains limited. Although the relationship of sex on health behaviors is a frequently studied area, few studies focus specifically on physiotherapy students.

Differences in health behaviors between men and women entail distinct potential health risks. Existing literature indicates that men are more likely than women to engage in unhealthy behaviors (Sok et al., 2020; El-Kader et al., 2023). Among negative health behaviors that distinguish men from women are smoking, with men smoking approximately 40% more than women. Alcohol consumption data similarly show higher intake among men, and the same trend applies to drug use frequency (Korpowicz, 2013). Our findings confirmed these data, showing that male physiotherapy students engage in significantly more unhealthy behaviors compared to female students. Further research is needed to precisely determine the mechanisms underlying this relationship, but educational programs on the harms of unhealthy behaviors should particularly target men in this group.

Health behaviors are closely related to personality traits (Willroth et al., 2021). Personality factors that predispose individuals to unhealthy behaviors include emotional immaturity, low stress tolerance, excessive dependence on others, difficulty in emotional expression, high anxiety, a sense of isolation, and low self-esteem (Ogińska-Bulik, 2010). Alongside typical personality traits, individual personal resources—strongly connected to personality—are also influential in shaping health behaviors. The study by Williams et al. has shown that individuals with Type D personality report fewer healthy behaviors (Williams et al., 2008), while Nowak et al. observed a correlation between Type D personality and poorer health behaviors among female dietetics students (Nowak et al., 2016). Our findings suggest that stress-prone personality traits may be associated with health behaviors in physiotherapy students. We observed that as the overall intensity of Type D personality and NE increased, levels of overall healthy behaviors, positive affect, and health practices decreased. Similarly, as SI increased, levels of healthy behaviors and positive affect declined. There is a lack of similar research examining how each dimension of Type D personality is related to health behaviors. Our findings suggest that SI may play a significant role in reducing the frequency of unhealthy behaviors. Considering that social inhibition reflects a tendency to suppress emotions and withdraw from others, further investigation with larger sample sizes is warranted.

The study found no statistically significant correlations between unhealthy behaviors and symptoms of musculoskeletal disorders, nor between healthy behaviors and symptoms of musculoskeletal disorders. The lack of a significant association between unhealthy lifestyles and musculoskeletal pain may be due to the participants’ very young age (average age 20). Furthermore, physiotherapy students, due to their field of study, may have knowledge of how to implement preventive measures to prevent musculoskeletal dysfunction. Therefore, the authors recommend conducting similar studies among physiotherapists with several years of professional experience.

Analyzing the relationship between Type D personality and musculoskeletal symptoms revealed that as NE increased, the number and overall severity of musculoskeletal pain symptoms also rose, expressed as the product of symptom intensity and frequency. Similar conclusions were drawn by Conden et al., who identified a strong association between Type D personality and psychosomatic symptoms and musculoskeletal pain, with adolescents with Type D personality reporting more symptoms (Condén et al., 2013). Adolescents with Type D personality had approximately twice the likelihood of experiencing musculoskeletal pain and five times the likelihood of psychosomatic symptoms. The NE subscale explained most of the association between Type D personality and psychosomatic and musculoskeletal symptoms (Condén et al., 2013). These findings, along with those of other authors, suggest that individuals with high levels of NE may be more susceptible to musculoskeletal disorders, which should be considered in clinical evaluations.

Our study also showed that as perceived stress and depressive symptoms increased, the levels of overall healthy behaviors, positive affect, and health practices decreased. Additionally, we found that as the intensity of Type D personality and its NE and SI dimensions increased, levels of perceived stress and depressive symptoms also rose. Nowak et al. reached similar conclusions, noting a correlation between Type D personality, poorer health behaviors, and depression among female dietetics students (Nowak et al., 2016). Individuals with this personality type are at increased risk of mental health disorders such as anxiety, post-traumatic stress disorder, phobias, and somatic disorders (O’Riordan et al., 2020; Mols and Denollet, 2010). The negative emotions characteristic of stress-prone personalities are closely linked to health through mechanisms such as the physiological effects of stress, greater exposure to stress, and unhealthy behaviors (Kupper and Denollet, 2018). Previous research by the authors of this study has shown that students with Type D personality are more likely to experience depression and higher levels of perceived stress (Gębska et al., 2021a; Gębska et al., 2021b). This relationship has been corroborated by other researchers (Howard and Hughes, 2012; Sharma, 2021; Sensoy and Sen, 2020).

In conclusion, the topic of evaluating health behaviors and Type D personality among physiotherapy students in the context of musculoskeletal disorders is insufficiently covered in the literature and requires further investigation. Based on current knowledge of these relationships, it is essential to educate physiotherapy students about the potential positive and negative impacts of health behavior choices to facilitate better decision-making. Our findings may be valuable for medical university administrators in developing initiatives that encourage physiotherapy students to adopt healthier lifestyles.

## Limitations

The results of this study should be interpreted with caution due to several key limitations: the cross-sectional nature of the study, the relatively small sample size, and the lack of a control group. The main limitation is the lack of a musculoskeletal physical examination, which would have allowed for a more objective assessment of disorders. Furthermore, the Beck Depression Inventory (BDI) score, used in this study for self-assessment of mood, is only indicative and does not constitute a formal diagnosis of depression. Another limitation was the narrow age range of the study participants and their young age. This may have contributed to the lack of a significant association between an unhealthy lifestyle and musculoskeletal pain. Therefore, the authors recognize the need for further research that would include a comprehensive musculoskeletal physical examination and utilize more rigorous psychological assessment tools.

## Conclusion

1. Promoting healthy behaviors among physiotherapy students is crucial, with attention to sex-specific differences.
2. Negative emotionality is associated with the frequency and intensity of musculoskeletal pain symptoms.
3. Type D personality is linked to the health behavior patterns observed in physiotherapy students.

## Data Availability

The original contributions presented in the study are included in the article/supplementary material, further inquiries can be directed to the corresponding author.
